# Editorial: Novel strategies for caries control

**DOI:** 10.3389/fcimb.2025.1756613

**Published:** 2025-12-19

**Authors:** Xuelian Huang, Andréa Ferreira Zandoná

**Affiliations:** Division of Restorative and Prosthetic Dentistry, The Ohio State University College of Dentistry, Columbus, OH, United States

**Keywords:** artificial intelligence (AI), caries control, dental biofilm, dental caries, novel strategies, post-antibiotic era, probiotics

Dental biofilm (plaque) is one of the primary contributors in the development of oral diseases, including dental caries, a “biofilm-mediated, sugar-driven, multifactorial, dynamic disease that poses significant public health concerns.” Dental caries is more than a localized oral health concern—it is intricately linked to systemic health, underscoring its significance as a broader public health issue. According to the caries ecology hypothesis, a sugar-driven imbalance in oral biofilms triggers caries development, making biofilm control and dietary interventions central to caries prevention. Despite extensive research and clinical practice targeting oral biofilms, current strategies remain insufficient. In the Post-Antibiotic Era, there is an urgent need for the development of novel strategies for caries control whether through direct targeting of oral biofilms or through modulation of the oral environment.”

This Research Topic assembles original research and reviews on novel caries control strategies, including: antibacterial agents and novel molecules (allulose, L-arginine, lysine acetylation), novel potential targets (fungi, particularly *Candida albicans*), advanced anti-biofilm dental materials (anti-caries hydrogels, zein-based curcumin nanocapsules, drug-delivery nanosystems), probiotics (*Lactobacillus salivarius*), and novel strategies to prevent secondary caries.

Han et al. evaluated the cariogenic potential of allulose using multi-tiered *in vitro* models, including single-species, dual-species, and saliva-derived biofilms. Compared to sucrose, glucose, and fructose, allulose significantly reduced bacterial growth, acid production, and biofilm formation, resembling non-fermentable sugar alcohols such as xylitol and erythritol. Biofilms grown with allulose lacked dense EPS-rich structures, while saliva-derived biofilms maintained higher microbial diversity with health-associated genera. These findings suggest that allulose has low cariogenic potential and may serve as a non-cariogenic, microbiome-friendly sugar alternative.

Gao et al. investigated the effects of L-arginine on key cariogenic oral microbes *Streptococcus mutans* and *C. albicans*. Using planktonic growth assays, biofilm biomass measurements, crystal violet staining, CFU counts, fluorescence *in situ* hybridization (FISH), and assessments of extracellular polysaccharide and lactic acid production in dual-species biofilms, they found that L-arginine inhibited both planktonic growth and biofilm formation in single-species and dual-species cultures, reduced biofilm adhesion, and suppressed extracellular polysaccharide and acid production. L-arginine may therefore represent a novel strategy to disrupt cross-kingdom interactions and synergistic cariogenicity.

Wang et al. reviewed secondary caries, a major cause of restoration failure, emphasizing the need for antimicrobial strategies in restorative materials. They categorized materials as releasing or non-releasing based on their antimicrobial mechanisms. Traditional release-based approaches often lack precision, durability, and adaptability for long-term caries prevention. The review focused on next-generation controlled-release antimicrobial systems, discussing the design of novel nanomaterials, their functional efficacy, and the mechanisms of representative antimicrobial agents. These advanced systems aim to provide sustained, targeted antimicrobial activity, enhancing restoration longevity and effectively inhibiting secondary caries.

Chen et al. summarized advances in anti-caries hydrogels for the prevention and treatment of dental caries. The review discussed natural and semi-synthetic polymers as hydrogel matrices and various hydrogel types, including probiotic, antibacterial, remineralization-inducing, and saliva-related caries-reducing hydrogels. Mechanisms, functional efficacy, current research status, and limitations were highlighted. These hydrogels show promise in modulating microbial dysbiosis, promoting remineralization, and providing targeted caries prevention, offering a versatile platform for future oral health therapeutics.

Erckmann et al. developed zein-based curcumin nanocapsules (Nano-curcumin) for minimally invasive caries management. Synthesized via nanoprecipitation, nanocapsules were characterized for size, morphology, encapsulation efficiency, release, and biocompatibility, and tested against *S. mutans* on dentin slices with or without photodynamic therapy (PDT). Nano-curcumin exhibited high encapsulation (~100%), spherical morphology, low polydispersity, sustained 24-hour release, and good cytocompatibility. Both Nano-curcumin and PDT-enhanced Nano-curcumin significantly reduced bacterial CFU, with PDT providing the greatest reduction, demonstrating their potential as safe and minimally invasive anti-caries agents.

Zhou et al. reviewed the role of fungi, particularly *Candida albicans*, in dental caries. Fungal biofilms release extracellular DNA (eDNA) and DNA-containing extracellular vesicles (EVs), which, together with bacterial eDNA, form the biofilm matrix and activate the host cGAS-STING signaling pathway. The review detailed molecular mechanisms of STING activation by viral, bacterial, and fungal DNA, explored direct and indirect fungal-mediated activation, and highlighted dual immune effects—enhancing antifungal defense while potentially promoting tissue damage via inflammation. Knowledge gaps were identified, with directions proposed for targeted prevention and treatment.

Yang et al. summarized the role of lysine acetylation, a post-translational modification, in regulating oral microbiota. Lysine acetylation influences bacterial metabolism, virulence, stress responses, and EPS production, affecting biofilm formation and colonization. For instance, acetylation of lactate dehydrogenase in *S. mutans* reduces acid production and tolerance, lowering cariogenic potential. Acetylation also enables bacterial adaptation to fluctuating oral environments, including hypoxia, and interacts with other PTMs to modulate protein function. Understanding these mechanisms offers insights into microbial adaptation and pathogenesis, providing potential therapeutic targets for oral disease prevention.

Ma et al. investigated the antibiofilm effects of *L. salivarius* supernatant on *S. mutans*. Biofilms were treated with cell-free supernatant, followed by RNA-seq, qRT-PCR, and non-targeted metabolomic analysis. The supernatant inhibited biofilm formation by suppressing phosphoenolpyruvate-dependent phosphotransferase systems, ATP-binding cassette transporters, two-component systems, quorum sensing, acid stress responses, and EPS production, without directly affecting glucosyltransferase genes. Metabolomic analysis identified active compounds, including phenyllactic acid, sorbitol, and honokiol, suggesting *L. salivarius* as a promising probiotic for caries prevention.

Du et al. reviewed nanosystems for drug delivery in dental caries management. Traditional drug delivery suffers from poor tissue penetration, short action duration, and low specificity. Nanosystems enhance drug stability, solubility, and bioavailability while minimizing side effects. Their roles include inhibiting bacterial survival, preventing biofilm formation, reducing demineralization, and promoting remineralization. These properties position nanosystems as next-generation strategies, with the potential to become mainstream approaches for caries prevention and treatment.

Collectively, these studies underscore cutting-edge achievements and outline future directions for caries prevention and management. Beyond biofilm control, another critical approach involves inhibiting demineralization and promoting remineralization. Therefore, developing novel strategies that integrate biofilm management with mineralization regulation represents a promising research direction. Dietary intervention constitutes a further key component in achieving effective caries management. Furthermore, advancements in artificial intelligence (AI) offer new opportunities, such as creating intelligent systems for caries risk assessment and early screening, and discovering novel antimicrobial agents like antimicrobial peptides, which could significantly contribute to caries control.

